# A Deep Learning Model for IMMP-Based Residual Disease Monitoring in AML with Monocytic Differentiation

**DOI:** 10.3390/diagnostics16081244

**Published:** 2026-04-21

**Authors:** Jing Ding, Huiying Qiu, Chunling Zhang, Weilin Liu, Xinyi Jin, Ting Xu, Zongyue Lu, Jiatao Lou, Huidan Li

**Affiliations:** 1 Department of Laboratory Medicine, Shanghai General Hospital, Shanghai Jiao Tong University School of Medicine, Shanghai 200080, China; laughing_410@163.com (J.D.);; 2 Department of Hematology, Shanghai General Hospital, Shanghai Jiao Tong University School of Medicine, Shanghai 200080, China; 3 Department of Clinical Practice, Hangzhou Zhiwei Information and Technology Co., Ltd., Hangzhou 311200, China; 4 Department of Artificial Intelligence, Hangzhou Zhiwei Information and Technology Co., Ltd., Hangzhou 311200, China

**Keywords:** acute myeloid leukemia, monocytic differentiation, deep learning, immature monocyte percentage, residual disease monitoring

## Abstract

**Background:** Acute myeloid leukemia (AML) with monocytic differentiation poses significant clinical challenges, including high relapse rates and chemotherapy resistance. Current morphological assessment is limited by inter-observer variability, low sensitivity, and inefficiency, especially for detecting low-level residual disease. This creates an urgent need for automated, objective tools to improve diagnostic consistency and monitoring. Artificial intelligence, particularly deep learning, offers potential for extracting high-dimensional cytomorphological features to address these gaps. **Methods:** A retrospective cohort of 184 bone marrow smear slides from patients with monocytic leukemia was used. The core biomarker was the immature monocyte percentage (IMMP), defined as monoblasts plus promonocytes among nucleated cells, with a 2.0% clinical cutoff. An EfficientNet-based convolutional neural network was developed via transfer learning and trained to classify four cell types: monoblasts, promonocytes, monocytes, and other cells. **Results:** The model achieved robust cell-level classification, with F1 scores of 0.82 for monoblasts and 0.34 for promonocytes. At the slide level, using an optimized IMMP threshold of 0.045, it accurately assessed persistent leukemic cell burden with 78.9% *Accuracy*, 81.1% *Recall*, and 76.9% *Specificity*. Model-predicted IMMP values showed strong correlation with expert-derived values (Pearson r = 0.827), demonstrating reliable quantitative agreement. **Conclusions:** This deep learning model provides an automated, objective tool for quantifying immature monocytes, addressing key limitations in morphological assessment of monocytic AML. The IMMP metric shows promise for monitoring treatment response, predicting relapse, and potentially identifying patients at risk of venetoclax-based therapy resistance. While promising, prospective multicenter validation is needed to translate these findings into routine clinical practice.

## 1. Introduction

Acute myeloid leukemia (AML) is a malignant clonal disorder of hematopoietic tissue characterized by the accumulation of abnormal (leukemic) blast cells, primarily in the bone marrow, and impaired production of normal blood cells [1,2]. Globally, AML accounts for ~30% of adult leukemias, with an age-adjusted incidence of 4.3 per 100,000 individuals [2]. Among AML subtypes, those with monocytic differentiation (French-American-British (FAB) M4/M5) represent 10–15% of cases, disproportionately affecting elderly patients (median age: 68 years) [3]. This subtype presents unique clinical hurdles: it exhibits a high relapse rate, a predilection for extramedullary manifestations (EMMs) (e.g., gingival infiltration, central nervous system involvement) and inherent resistance to conventional chemotherapy [4,5]. Despite recent advances in targeted therapy (e.g., FLT3 inhibitors) and stem cell transplantation, the 5-year overall survival (OS) rate for patients with AML with monocytic differentiation remains below 40% [6], underscoring an urgent need for more precise diagnostic tools and dynamic monitoring strategies to improve clinical outcomes.

Currently, the diagnosis and post-treatment monitoring of AML with monocytic differentiation face multiple challenges. Morphological examination, while fundamental for diagnosis, is time-consuming, labor-intensive, and inefficient. Its accuracy and reliability are further limited by the morphological heterogeneity of immature monocytic cells after treatment and inter-observer variability, especially when the residual cell proportion is low (2–5%), leading to potential misjudgment [7,8,9]. Flow cytometric assessment, although important in AML, may be confounded in this subtype by phenotypic heterogeneity, antigenic drift after therapy, and the difficulty of distinguishing residual leukemic monocytic precursors from regenerating precursors or reactive monocytosis [10,11]. Collectively, these gaps lead to suboptimal risk stratification and delayed relapse intervention—key drivers of poor survival. Therefore, there is an urgent clinical demand for auxiliary technologies that can enhance the consistency, sensitivity, and efficiency of morphological assessment.

In recent years, artificial intelligence (AI) has advanced rapidly and been increasingly applied to disease diagnosis and treatment, particularly in the analysis of medical images [12]. Beyond its widespread use in medical radiology, AI has also garnered growing attention in the field of cellular image analysis [13]. Deep learning (DL) models, especially convolutional neural networks (CNNs), have emerged as powerful tools for extracting high-dimensional cytomorphological features from large datasets, reducing human bias, and improving the sensitivity of detecting low-abundance cells. For instance, Chen et al. introduced an AI-based method with high sensitivity for detecting circulating plasma cells (CPC), enabling early and accurate CPC identification through morphological examination [14]. Although DL models have been widely adopted for bone marrow cell recognition and are trained on comprehensive databases encompassing all major cell types, they continue to demonstrate significant limitations in accurately identifying monocytes [15,16]. This gap is particularly critical in the context of AML with monocytic differentiation, where monocytes and promonocytes—collectively termed the IMMP—serve as essential markers for post-treatment monitoring. While prior research has applied CNNs to classify monocytes in peripheral blood smears [8], no study to date has leveraged transfer learning to enhance the recognition of immature monocytes in bone marrow specimens. Furthermore, the potential of IMMP as a dynamic, biologically informed surrogate marker for disease stratification remains unexplored in computational models.

To address these gaps, we developed a CNN-based automated bone marrow cell recognition system to improve classification accuracy of immature monocytic cells in AML with monocytic differentiation and to establish an IMMP-based slide-level assessment of disease burden. Using a retrospectively annotated dataset, we trained and validated a model to evaluate its performance in detecting persistent leukemic cells during post-treatment monitoring. This approach seeks to provide an automated, objective tool to support precise diagnosis, treatment evaluation, and long-term management of monocytic-differentiated AML.

This study aims to develop an AI-assisted diagnostic and monitoring system for AML with monocytic differentiation to improve the automated recognition accuracy of immature monocytic cells and to construct a dynamic post-treatment prediction model for early relapse warning. The research seeks to provide a novel paradigm for the precise diagnosis, treatment, and long-term management of AML with monocytic differentiation, ultimately improving patient survival quality and reducing the healthcare burden.

## 2. Materials and Methods

### 2.1. Patient Cohort and Sample Stratification

This retrospective study included 184 bone marrow smear slides derived from 90 patients who were diagnosed with acute monocytic leukemia (AMoL) and 8 patients who were diagnosed with chronic myelomonocytic leukemia (CMML) according to the French-American-British (FAB) classification [17]. All samples were sourced from the Department of Hematology, Shanghai First People’s Hospital. The smears were originally prepared during routine diagnostic evaluations (counting 200 cells per slide) [18], and their dates of acquisition ranged from 1 June 2017 to 31 December 2024.

To ensure data quality and consistency, all bone marrow smears included in the analysis met the following criteria: (1) confirmed AMoL/CMML diagnosis at the time of initial disease evaluation; (2) adequate Liu’s staining (BaSO Biotech Co., Ltd., Zhuhai, China; catalog no. BA4001) quality suitable for digital scanning and cytomorphological assessment; (3) availability of complete clinical and laboratory records for retrospective data verification.

To support the development of a morphological analysis model for monitoring post-treatment relapse in AMoL, each smear was analyzed for its IMMP, defined as the proportion of immature monocytes (monoblasts plus promonocytes) among all bone marrow nucleated cells. In this study, 2.0% was adopted as the study-defined cut-off for the proportion of immature monocytes, in line with the upper limit of the generally accepted normal bone marrow range, where blast counts typically remain below 2.0%, and consistent with routine laboratory practice in which 2.0% is commonly used as a clinically meaningful alert threshold to indicate abnormal expansion of immature cells [19]. Based on this threshold, IMMP further served as a biological surrogate reflecting different disease states of AMoL. An IMMP < 2.0% were considered negative and were generally obtained from patients previously diagnosed with AMoL who had achieved post-treatment morphological recovery with normalization of bone marrow findings. In contrast, Smears with 2.0% ≤ IMMP < 5.0% still fulfilled the criteria for morphological remission according to the European LeukemiaNet (ELN) standards, although they were frequently associated with low-level residual disease and were often derived from patients who subsequently relapsed [20,21]. By comparison, IMMP ≥ 5.0% were predominantly collected at the time of initial AMoL presentation, corresponding to a high leukemic cell burden [10]. Given that the system is designed to detect persistent leukemia cells in post-treatment patients (who typically exhibit low IMMP values), the test cohort was confined to smears with an IMMP of <20.0%. This additional criterion ensured that cases refractory to induction chemotherapy (which retain over 20.0% leukemia cells) were excluded from the testing cohort. Thereby allowing the model evaluation to be specifically focused on the clinically relevant, low-IMMP follow-up population.

Following the study-defined IMMP categories, all 184 smears were divided into a training cohort (*n* = 108) and an independent testing cohort (*n* = 76). The partitioning was performed while preserving the relative distribution of the two IMMP-defined levels of immature monocytic cell burden, ensuring that both cohorts contained representative samples across the full IMMP spectrum. Following cohort assignment, all smears were digitized using the Morphogo system (Hangzhou Zhiwei Information Technology Co., Ltd., Hangzhou, China), which provided whole-slide imaging (WSI) and automated bone marrow nucleated cell localization and classification to support downstream IMMP computation and analyses related to persistent leukemic cell burden.

### 2.2. Digitization Workflow and Dataset Construction

All bone marrow smears were digitized using the Morphogo. Each slide was first scanned at 40× magnification to generate a complete WSI. The system then automatically identified the region most suitable for cytological assessment and captured 500 bone marrow nucleated cells at 100× oil-immersion high resolution for subsequent processing.

A DL dataset was then constructed from the extracted cell images. The training cohort comprised 108 smears, including 15 slides with IMMP < 2.0%, 8 slides with 2.0% ≤ IMMP < 5.0%, and 85 slides with IMMP ≥ 5.0%, yielding 91,030 bone marrow nucleated cell images. Among these, 33,419 were monoblasts, 7627 promonocytes, 6285 monocytes, and 43,699 other bone marrow nucleated cells. All cell images were independently reviewed and annotated by two senior hematopathologists, with discrepancies adjudicated by a third reviewer. Following annotation, the training cohort was randomly partitioned, with 80% of the images used for model training and 20% reserved for validation. The validation cohort was used to evaluate intermediate model performance during algorithm development, enabling assessment of classification accuracy after each training iteration and helping identify category-specific weaknesses. These validation results guided targeted sample supplementation and model refinement across multiple training cycles until satisfactory and stable classification performance was achieved.

The testing cohort consisted of 76 smears, including 39 slides with IMMP < 2.0%, 17 slides with 2.0% ≤ IMMP < 5.0%, and 20 slides with 5.0% ≤ IMMP < 20.0% from which 42,838 bone marrow nucleated cell images were obtained. All images in the testing cohort were completely independent from those used for model development and were processed by the trained model to evaluate generalization performance. Model-generated counts of immature monocytes were subsequently used to calculate the slide-level IMMP, which served as the basis for assessing persistent leukemic cell burden.

### 2.3. DL Model Development Using the Strategy of Transfer Learning

An EfficientNet-based CNN was developed to perform automated morphological classification of bone marrow nucleated cells extracted from digitized bone marrow smears. Before training, all cell images underwent standardized preprocessing to ensure uniformity and robustness. Each image was center-cropped to remove excessive background, resized to the input resolution required by EfficientNet, and subjected to color normalization to minimize staining variability [22]. Illumination correction was applied to reduce brightness inhomogeneity caused by oil-immersion microscopy, and images affected by blur, scanning artifacts, or incomplete cell boundaries were automatically excluded [23]. To enhance generalization ability, data augmentation was applied during training, including random rotation, flipping, and mild color jitter [24].

After preprocessing, the cell images were fed into the EfficientNet-B2 backbone (the proprietary Morphogo base model) as a feature extractor, which extracted multi-scale morphological features through its compound scaling of network depth, width, and resolution. The hierarchical feature outputs were integrated via weighted feature fusion to capture both fine-grained cytological structures and higher-level semantic patterns [25]. A linear classifier head was attached to the final feature representation to output probabilistic predictions for the task-specific target classes.

To improve model adaptability for immature monocytic cell classification, a transfer learning strategy was utilized. The Morphogo EfficientNet-B2 base model was previously trained for 300 epochs on a large-scale cohort of 9 million annotated bone marrow cells comprising 35 morphological classes [15]. The backbone was initialized with weights from this general-purpose bone marrow cell classification model trained on the Morphogo internal cell image repository. These pretrained weights provided broadly representative cytomorphologic features. Following initialization, three classification variants were implemented by modifying the linear classifier head on the pre-trained EfficientNet-B2 backbone: a full 35-class classifier, a binary classifier (immature monocytes vs. other), and a 4-class classifier (monoblasts, promonocytes, mature monocytes, and others). The model was then fine-tuned on the monocyte-focused dataset used in this study, during which only the upper network layers and the final classification head were updated, allowing for efficient adaptation to the specific task while retaining generalizable low-level feature representations. All models were fine-tuned for 100 epochs with the AdamW optimizer and a learning rate of 1 × 10^−4^. This approach allowed the models to leverage the underlying morphological features learned from the 9 million bone marrow cell images while adapting to specific monocyte differentiation stages (all original code has beenwas deposited at Github: https://github.com/zongyue-lu/monocyte-study accessed on 25 February 2026).

To stabilize convergence and reduce overfitting, we applied regularization strategies such as dropout and weight decay and continuously monitored validation performance during training [25].

### 2.4. Performance Evaluation

Three hematopathologists independently reviewed the cell recognition results and evaluated the performance of the DL model in classifying bone marrow nucleated cells and identifying monocytic cells. To minimize subjective variability, the cell classification interpretation was considered valid only when at least two of the three experts reached consensus. These expert-reviewed classifications served as the gold standard and were compared with the model-generated predictions.

A multi-class confusion matrix was constructed to visualize classification performance across monoblasts, promonocytes, monocytes, and other bone marrow nucleated cells, allowing for direct assessment of misclassification patterns within the monocytic lineage. In addition, t-Distributed Stochastic Neighbor Embedding (t-SNE) visualization of the model’s feature representations demonstrated clear separation among the three monocytic subpopulations, indicating effective extraction of stage-specific morphological features.

For slide-level assessment of persistent leukemic cell burden, cases confirmed as positive by expert morphological review were considered the reference standard. Among these, slides correctly identified as positive by the algorithm were defined as true positives (*TP*), whereas those misclassified as negative were defined as false negatives (*FN*). Conversely, among negative cases, slides correctly identified as negative were defined as true negatives (*TN*), and those incorrectly predicted as positive were defined as false positives (*FP*). The model’s performance in assessing persistent leukemic cell burden was evaluated using *Recall*, *Specificity*, *Accuracy*, positive predictive value (*PPV*), negative predictive value (*NPV*), and *F1-score*, calculated on the basis of *TP*, *FN*, *TN*, and *FP* as defined above. The calculation method is as follows:

*Recall = (TP/(TP + FN))* × 100% (1)

*Specificity = (TN/(TN + FP))* × 100% (2)

*Accuracy = ((TP + TN)/(TP + TN + FP + FN))* × 100% (3)

*PPV = (TP/(TP + FP))* × 100% (4)

*NPV = (TN/(FN + TN))* × 100% (5)

*F1 score =* 2 *× PPV × Sensitivity/(PPV + Sensitivity)* (6)

These metrics reflect the model’s ability to distinguish smears with positive versus negative persistent leukemic cell burden through automated estimation of the IMMP, which depends on the recognition of monoblasts and promonocytes. To further visualize the discriminative ability of the machine-selected IMMP threshold, a slide-level confusion matrix was generated, providing an intuitive assessment of the algorithm’s capacity to differentiate smears with positive and negative persistent leukemic cell burden.

In addition to threshold-based classification, a quantitative agreement analysis was performed to evaluate the relationship between model-predicted IMMP values and expert-derived IMMP values at the slide level. Expert-derived IMMP values were calculated based on hematopathologist-guided classification of bone marrow nucleated cells. Pearson and Spearman correlation analyses were conducted to assess linear correlation and rank-order consistency, respectively. Linear regression analysis was further performed to characterize the overall relationship between predicted and expert-derived IMMP values. *FP* and *FN* cases were identified based on the selected decision threshold and visualized in the scatter plot to examine their distribution relative to the decision boundaries.

## 3. Results

### 3.1. Performance of Monocytic Cell Recognition

**Figure 1:**
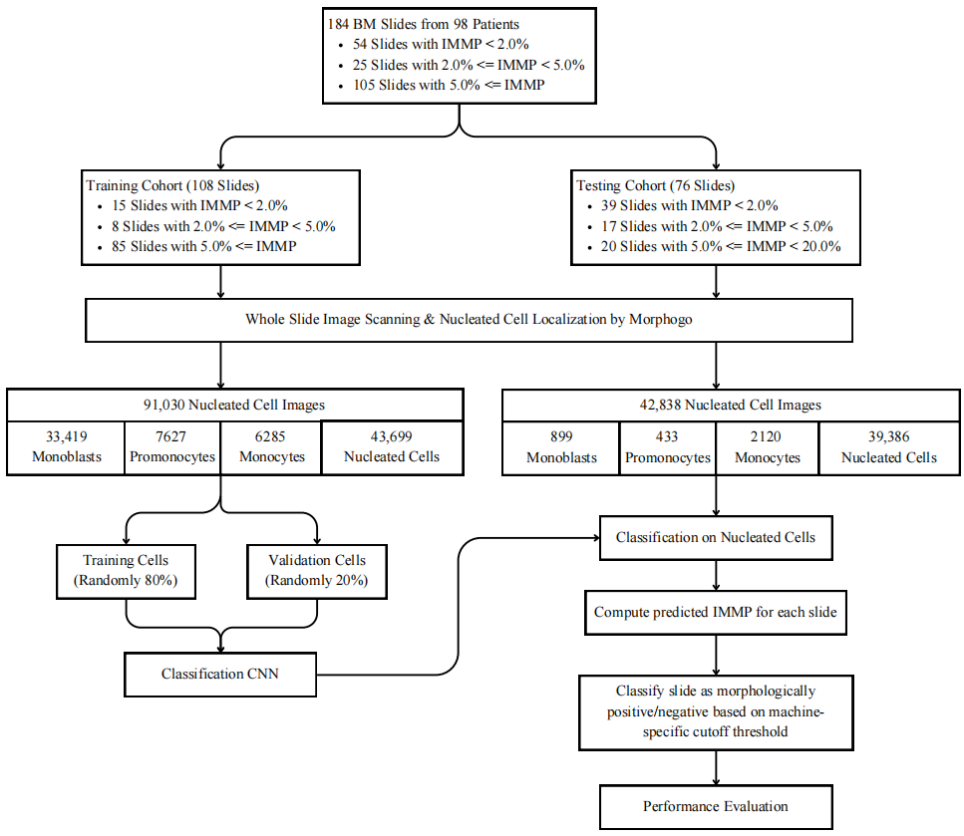
Workflow of IMMP-based dataset construction, bone marrow nucleated cells classification, and assessment of persistent leukemic cell burden using the Morphogo digital pathology system. Slides were stratified into three study-defined IMMP categories (IMMP < 2.0%, 2.0% ≤ IMMP < 5.0%, and IMMP ≥ 5.0%) reflecting different levels of immature monocytic cell burden. Class proportions were preserved across the training (*n* = 108) and testing (*n* = 76) cohorts to ensure balanced representation for model development and evaluation. All smears underwent whole-slide imaging and automated bone marrow nucleated cell localization and classification using the Morphogo system, after which predicted immature monocyte counts were used to calculate slide-level IMMP for assessing persistent leukemic cell burden. IMMP: immature monocyte percentage (percentage of monoblasts and promonocytes among all nucleated cells).

Accurate recognition of monocytic cells at different maturation stages is a foundational step for reliable calculation of the IMMP, which directly underpins the assessment of persistent leukemic cell burden in post-treatment monitoring. The overall workflow of our study, from dataset construction based on IMMP stratification to slide-level assessment of residual disease, is illustrated in Figure 1.

**Figure 2:**
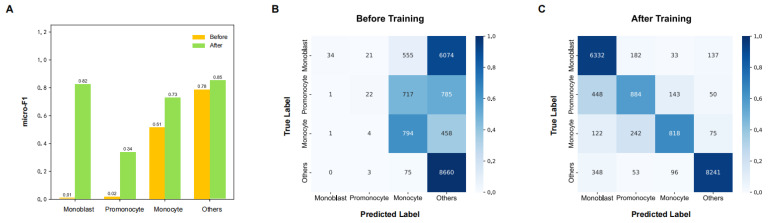
Monocytic cell recognition performance before and after model training. (**A**) Micro-F1 scores for monoblasts, promonocytes, monocytes, and other bone marrow nucleated cells before and after incorporation of study-annotated monocytic training samples. (**B**,**C**) Confusion matrices showing cell-level classification results for monoblasts, promonocytes, monocytes, and other bone marrow nucleated cells before (**B**) and after (**C**) model training.

At the cell level, the DL model demonstrated substantial improvements in identifying monocytic subpopulations after training on our study-specific annotated samples, as shown in Figure 2. Compared with the baseline initialization state, incorporation of the study-specific annotated training samples led to marked improvements in identifying monocytic subpopulations, particularly the two immature monocytic categories directly contributing to IMMP calculation, namely monoblasts and promonocytes. The micro-F1 scores increased to 0.82 for monoblasts and 0.34 for promonocytes, while monocyte recognition reached 0.73 (Figure 2A). Consistently, the confusion matrices demonstrated a substantial reduction in misclassification between immature monocytic cells and other bone marrow nucleated cell categories after training, indicating improved discrimination across different stages of monocytic differentiation (Figure 2B,C).

**Figure 3:**
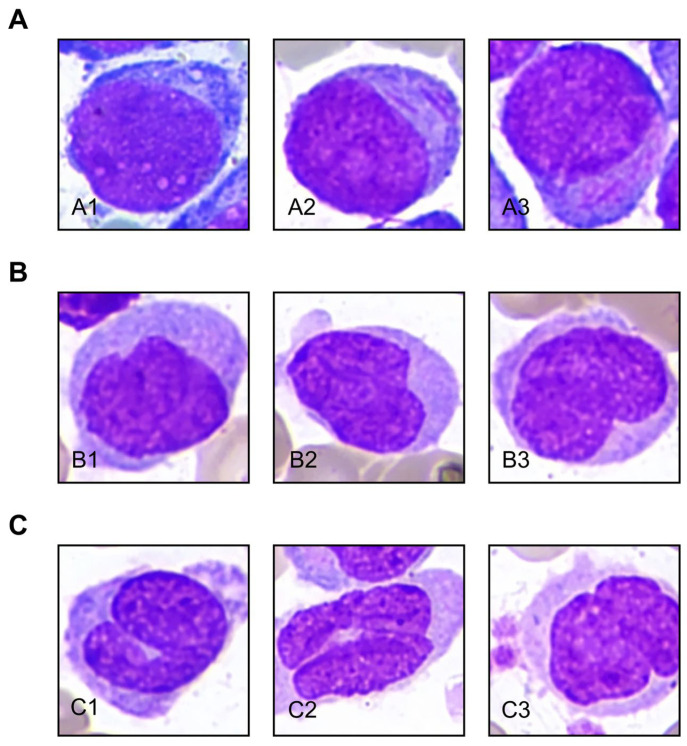
Representative morphological examples of monocytic cells across different stages of differentiation recognized by the trained model. (**A**) (**A1**–**A3**) monoblasts; (**B**) (**B1**–**B3**) promonocytes; (**C**) (**C1**–**C3**) monocytes.

Representative cell images illustrate the improved recognition of monocytic cells at different stages of differentiation, including machine-identified monoblasts (Figure 3A, A1–A3), promonocytes (Figure 3B, B1–B3), and monocytes (Figure 3C, C1–C3). These examples highlight the model’s enhanced ability to capture subtle morphological differences along the monocytic maturation continuum.

To quantitatively characterize these changes, *Recall*, *Specificity*, *Accuracy*, *PPV*, and *NPV* were calculated for monoblasts, promonocytes, and monocytes before and after training (Table 1). These improvements provide a more reliable cell-level basis for quantifying immature monocytic burden and strengthen the robustness of IMMP estimation in post-treatment samples, where residual disease is often subtle and the accurate enumeration of immature monocytic cells serves as a cornerstone for monitoring treatment response and predicting relapse in AMoL.

### 3.2. t-SNE Visualization of Feature Representation Before and After Model Training

**Figure 4:**
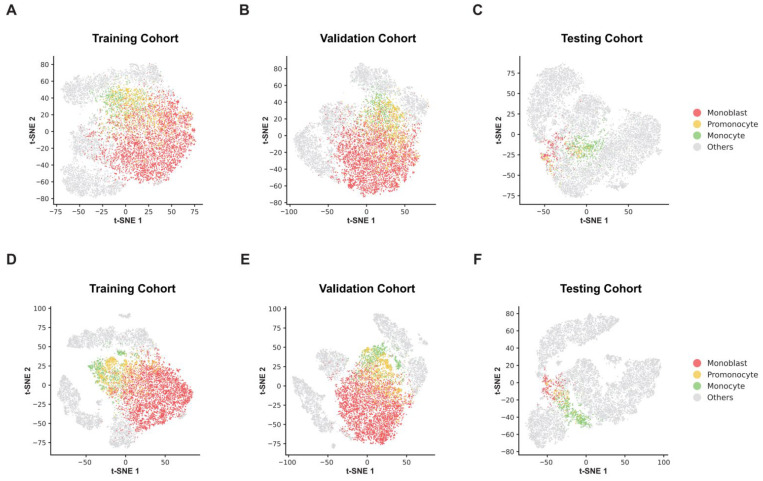
t-SNE visualization of monocytic cell feature representations before and after model training. (**A**–**C**) show feature embeddings prior to model training in the training (**A**), validation (**B**), and testing (**C**) cohorts, whereas (**D**–**F**) display feature embeddings after model training in the corresponding cohorts.

To visualize the evolution of feature representations learned by the DL model, t-SNE was applied to cell-level feature embeddings before and after model training across the training, validation, and testing cohorts (Figure 4). Prior to training, monoblasts, promonocytes, and monocytes exhibited substantial overlap in the embedded feature space, with poorly defined boundaries between different stages of monocytic differentiation and partial intermixing with other bone marrow nucleated cell populations (Figure 4A–C).

After model training, feature separability was markedly improved. In the training and validation cohorts, monoblasts, promonocytes, and monocytes formed more compact and distinguishable clusters, with reduced overlap among different stages of monocytic differentiation. Notably, promonocytes consistently occupied an intermediate feature space between monoblasts and monocytes, reflecting their transitional morphological characteristics (Figure 4D,E). Importantly, similar clustering patterns were preserved in the independent testing cohort, supporting the generalizability of the learned feature representations (Figure 4F).

Collectively, these results demonstrate that model training enhanced the discrimination of stage-specific monocytic morphological features, providing a robust feature basis for subsequent IMMP estimation and assessment of persistent leukemic cell burden.

### 3.3. IMMP-Based Threshold Selection and Performance Evaluation for Assessing Persistent Leukemia Cell Burden

Following the improved feature separability observed in the t-SNE analysis, we evaluated the ability of model-derived IMMP values to discriminate slide-level persistent leukemia cell burden. Slide-level burden status was defined by hematopathologist morphological review, using an IMMP value 2.0% as a reference boundary to distinguish smears with negative versus positive persistent leukemic cell burden.

**Figure 5:**
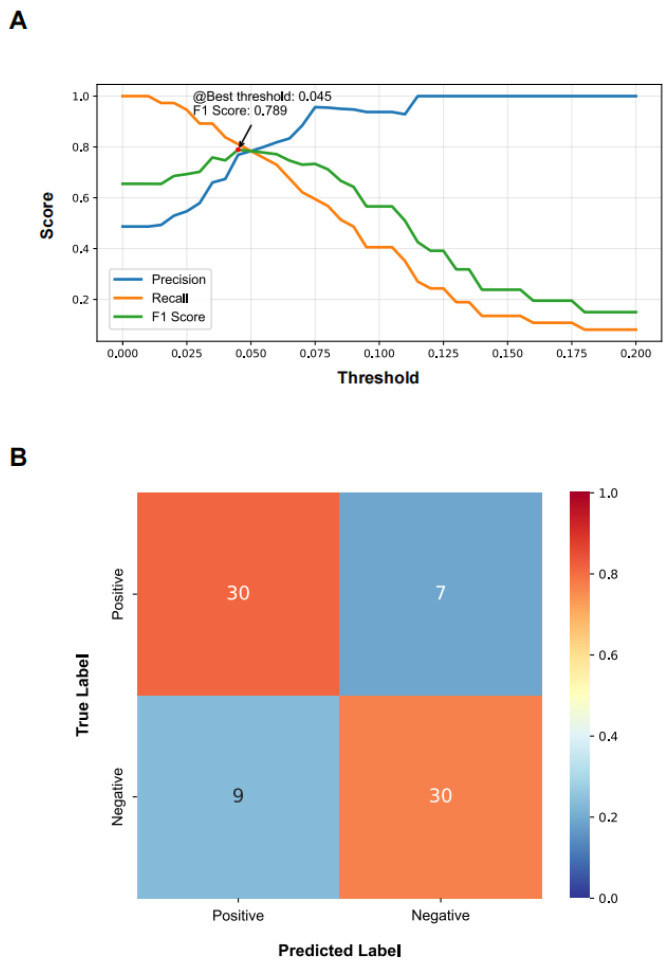
IMMP decision threshold selection and performance evaluation for assessing persistent leukemic cell burden. (**A**) Precision, *Recall*, and F1 score plotted across a range of IMMP decision thresholds to guide threshold selection for slide-level classification of persistent leukemic cell burden. (**B**) Confusion matrix summarizing slide-level classification performance at the selected IMMP threshold of 0.045, based on hematopathologist-defined assessment of persistent leukemic cell burden. IMMP: immature monocyte percentage.

Model performance was evaluated across a range of IMMP decision thresholds, with precision, *Recall*, and F1-score calculated at each threshold (Figure 5A). As the decision threshold increased, precision showed a progressive increase whereas *Recall* decreased, reflecting the expected trade-off between *FP* and *FN* predictions. The F1-score reached a maximum value of 0.789 at an IMMP decision threshold of 0.045, which was therefore selected as the final decision threshold for subsequent analyses.

Using this selected threshold, model performance was evaluated using a slide-level confusion matrix (Figure 5B). Among the 39 post-treatment bone marrow smears classified as negative for persistent leukemic cell burden by expert morphological review, 30 were correctly classified, while 9 were incorrectly predicted as positive. Among the 37 smears classified as positive for persistent leukemic cell burden, 30 were correctly classified, with 7 cases incorrectly predicted as negative. Based on these results, the model achieved an overall *Accuracy* of 0.789, with a *Recall* of 0.811, a *Specificity* of 0.769, a *PPV* of 0.769, and a *NPV* of 0.811 for assessing persistent leukemic cell burden (Table 2).

Together, these results demonstrate that IMMP-based thresholding enables effective slide-level classification of persistent leukemia cell burden with balanced sensitivity and *Specificity*.

### 3.4. Quantitative Agreement Between Model-Predicted and Expert-Derived IMMP Values

**Figure 6:**
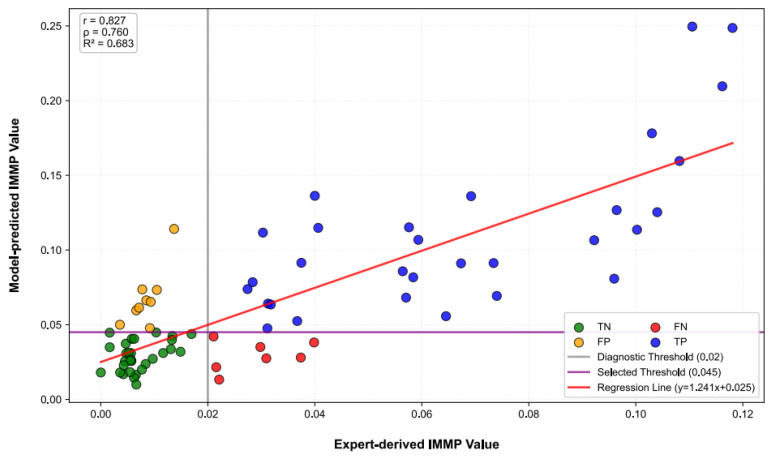
Quantitative agreement between model-predicted and expert-derived IMMP values for assessing persistent leukemia cell burden. Scatter plot showing the relationship between model-predicted IMMP value (*y*-axis) and expert-derived IMMP value (*x*-axis), which were calculated based on hematopathologist-guided classification of bone marrow nucleated cells, across the testing cohort (*n* = 76). The solid red line indicates the linear regression fit, while the vertical and horizontal lines denote the expert-defined IMMP reference boundary for persistent leukemic cell burden (0.02) and the selected model threshold (0.045), respectively. Data points are color-coded according to classification outcomes, including *TP* (*n* = 30), *TN* (*n* = 30), *FP* (*n* = 9), and *FN* (*n* = 7). Pearson and Spearman correlation coefficients, as well as the coefficient of determination (R^2^), are shown. IMMP: immature monocyte percentage; *TP*: true positives; *TN*: true negatives; *FP*: false positives; *FN*: false negatives.

To further assess the agreement between model-predicted IMMP value and hematopathologist-derived IMMP value, a correlation analysis was performed comparing predicted and expert-derived IMMP values across the testing cohort (Figure 6). A strong positive correlation was observed (Pearson r = 0.827; Spearman ρ = 0.760), with a linear regression explaining 68.3% of the variance (R^2^ = 0.683), indicating that the model preserves meaningful continuous information related to persistent leukemia cell burden. Notably, most *FP* and *FN* cases were distributed near the decision boundaries, suggesting that misclassifications predominantly occurred in borderline samples.

Collectively, these data indicate that the selected IMMP threshold provides reliable and balanced slide-level classification performance in post-treatment samples, supporting the feasibility of IMMP-based automated assessment of persistent leukemia cell burden for detecting subtle residual disease and for identifying patients with no morphological evidence of relapse.

## 4. Discussion

AML is an aggressive hematologic malignancy, and intensive chemotherapy is often poorly tolerated by elderly or medically unfit patients [26,27], for whom venetoclax combined with hypomethylating agents has become a standard treatment with significantly improved clinical outcomes [28,29,30]. However, responses to venetoclax-based regimens vary by leukemic differentiation state: primitive AML is typically more sensitive, while AML with monocytic differentiation shows relative resistance [31,32,33]. This resistance is linked to monocytic leukemia stem cells, which differ from primitive leukemia stem cells in immunophenotype, transcriptional programs, and metabolic dependencies [31,34]. Therefore, accurate identification of monocytic-differentiated leukemic cells is clinically relevant for diagnosis, disease monitoring, treatment stratification, and resistance assessment.

In the present study, we developed and validated a deep learning-based AI model for automated bone marrow cell morphology analysis. After specialized training, the model demonstrated significant improvements in cell type recognition: the F1-score for monocyte identification increased from 0.0101 to 0.9089, and for promonocyte identification from 0.0279 to 0.6126. Based on these predictions, we propose the IMMP as a quantitative metric, translating the biological phenotype of monocytic differentiation into an objective clinical parameter. In an independent test set (*n* = 76), an IMMP threshold of 0.045 showed robust diagnostic performance, with an F1-score of 0.7895, *Accuracy* of 78.95%, sensitivity of 81.08%, and precision of 76.92%. Together, these findings indicate that the model can capture clinically meaningful morphological information and translate it into a quantitative parameter for post-treatment monitoring.

Prior work has consistently demonstrated the inherent difficulty of monocytic precursor recognition: general blast detection models such as BMSNet achieved only 0.61 *Accuracy* for monoblast identification, far lower than the >0.80 *Accuracy* for non-monocytic myeloid blasts [16]; peripheral blood classification models similarly reported 15–20% lower *Accuracy* for promonocytes compared to mature cell types [35]; and even inter-observer agreement among expert hematopathologists for monocytic cell classification is only 0.81 ± 0.07 [8], highlighting the high subjectivity of this task. Against this background, our model achieves 0.89 *Accuracy* for promonocyte classification and a Pearson correlation of 0.827 with expert-derived IMMP counts, representing a meaningful improvement for this specialized clinical use case.

A key consideration is the interpretation of IMMP thresholds in this study. The 2.0% threshold is a clinically grounded reference standard, aligned with routine morphological practice and the conventional upper limit of immature monocytic precursors in normal bone marrow assessment. In contrast, the 0.045 threshold identified in our analysis is a model operating threshold (not a new clinical diagnostic cutoff), selected to maximize the F1-score in the test cohort and reflect the algorithm’s calibration characteristics. The discrepancy between these two thresholds likely stems from residual classification errors, particularly in promonocyte identification. Despite this, the strong correlation between model-predicted and expert-annotated IMMP values confirms the biological validity of the model output, indicating the algorithm retains the underlying biological signal even with imperfect numerical calibration.

The potential clinical value of this model extends beyond simple automation of morphology review. First, IMMP may serve as a practical quantitative biomarker for identifying patients with persistent immature monocytic burden after treatment, thereby supporting earlier detection of morphologic relapse or residual disease. Second, because monocytic differentiation has been linked to reduced sensitivity to venetoclax/azacitidine, pre-treatment IMMP assessment may help identify patients at higher risk of primary resistance [31]. In this context, IMMP could become a readily available adjunctive parameter for treatment planning, particularly in elderly or unfit patients for whom therapeutic options are limited [36,37]. Third, the model provides a bridge between routine morphology and the emerging biological framework of leukemia stem cell heterogeneity [34]. By quantifying features associated with monocytic differentiation, it may complement genomic and immunophenotypic assays in a more integrated disease-monitoring strategy.

This study also has several limitations. First, the study was retrospective and based on samples from a single center, which may limit the generalizability of the findings. External validation using multi-center datasets with different staining conditions, scanners, and patient populations will be necessary. Second, the cell-level F1 score for promonocytes was relatively modest at 0.34, driven by two synergistic factors: biologically, as a transitional stage, promonocytes show substantial morphological overlap with monoblasts and mature monocytes, causing inherent ambiguity; methodologically, their scarcity and class imbalance in training data limited discriminative feature learning [8,9]. Third, although the slide-level results were encouraging, the sample size of the independent test cohort remained modest. Fourth, the current study focused on morphology-based assessment and did not integrate molecular, flow cytometric, or treatment-response data into a unified predictive framework. Therefore, the present findings should be interpreted as proof of concept for an AI-assisted diagnostic tool rather than as a replacement for established multimodal assessment.

Future studies should focus on: prospective multi-center validation of IMMP’s robustness and transportability; direct testing of its predictive value for venetoclax/azacitidine response in clinical cohorts; improving promonocyte recognition via larger annotated datasets, balanced sampling, and multimodal design; and integrating IMMP with cytogenetic, molecular, immunophenotypic, and longitudinal clinical data to enhance MRD assessment and post-treatment risk stratification in AML with monocytic differentiation.

## 5. Conclusions

Our deep learning model represents a significant breakthrough in the diagnosis and monitoring of AML. By providing an objective, reproducible, and sensitive method for quantifying immature monocytes, this tool effectively addresses critical unmet needs in the management of AML with monocytic differentiation. The IMMP metric offers a practical and cost-effective approach to identify patients at risk of venetoclax resistance and monitor treatment responses, with the potential to improve clinical outcomes. Prospective studies and technological optimization will be essential to translate these findings into routine clinical practice and advance the goal of personalized precision medicine for AML.

## Figures and Tables

**Table 1:** The performance of DL model for monocytic cell prediction.

Stage	Cell Type	*Recall*	*Specificity*	*Accuracy*	*PPV*	*NPV*
Before training	Monoblast	0.005	1.000	0.635	0.944	0.634
	Promonocyte	0.014	0.998	0.916	0.440	0.917
	Monocyte	0.632	0.921	0.901	0.371	0.971
After training	Monoblast	0.974	0.920	0.930	0.873	0.968
	Promonocyte	0.580	0.971	0.939	0.650	0.962
	Monocyte	0.651	0.984	0.961	0.750	0.974

*PPV*: positive predictive value; *NPV*: negative predictive value.

**Table 2:** The performance of DL model for assessing persistent leukemic cell burden.

Model	*Recall*	*Specificity*	*Accuracy*	*PPV*	*NPV*
DL model	0.811	0.769	0.789	0.769	0.811

*PPV*: positive predictive value; *NPV*: negative predictive value.

## Data Availability

The data presented in this study are openly available in Github at https://github.com/zongyue-lu/monocyte-study, accessed on 25 February 2026.

## Abbreviations

The following abbreviations are used in this manuscript:

AML	Acute Myeloid Leukemia
IMMP	Immature Monocyte Percentage
FAB	French-American-British
EMMs	Extramedullary Manifestations
OS	Overall Survival
AI	Artificial Intelligence
DL	Deep Learning
CNN	Convolutional Neural Network
MDS	Myelodysplastic Syndromes
CPC	Circulating Plasma Cells
AMoL	Acute Monocytic Leukemia
CMML	Chronic Myelomonocytic Leukemia
ELN	European LeukemiaNet
WSI	Whole-Slide Imaging
t-SNE	t-Distributed Stochastic Neighbor Embedding
TP	True Positives
FN	False Negatives
TN	True Negatives
FP	False Positives
PPV	Positive Predictive Value
NPV	Negative Predictive Value
HMAs	Hypomethylating Agents
LDAC	Low-Dose Cytarabine
m-LSCs	Monocytic Leukemia Stem Cells
p-LSCs	Primitive Leukemia Stem Cells
FCM	Flow Cytometry
LAIPs	Leukemia-Associated Immunophenotypes
MRD	Minimal/Measurable Residual Disease
VEN/AZA	Venetoclax/Azacitidine
